# Preliminary study on the explosive performance of coal dust and prospects for engineering applications

**DOI:** 10.1038/s41598-025-85274-x

**Published:** 2025-01-04

**Authors:** Jing Guo, Shirong Ge

**Affiliations:** China University of Mining and Technology (Beijing), Beijing, 100083 China

**Keywords:** Coal, Detonation characteristics, Detonation efficiency, Geochemistry, Mineralogy

## Abstract

This study aims to evaluate the efficiency and energy release characteristics of different types of coal in pulse detonation engines (PDE) to advance the development of deep coal fluidization detonation technology, achieving more efficient and cleaner coal utilization. Using a custom PDE setup, experiments were conducted with four coal types at mass flow rates from 30 to 120 g/s. High-frequency pressure sensors assessed pressure dynamics and detonation wave propagation, complemented by numerical simulations for accuracy. Results show that the maximum detonation pressure (P_max_) increases linearly with coal mass flow, with anthracite reaching 1.52 MPa at 120 g/s. A consistent linear relationship between Pmax and maximum temperature (*T*_max_) was observed. Detonation combustion efficiency (*η*_DCE_) improved with mass flow, with peat rising from 52.3% to 83.7%. These findings provide a foundation for advancing deep coal fluidized detonation technology, contributing to more efficient and cleaner coal utilization.

## Introduction

Coal, as a critical energy resource, plays an indispensable role in global industrial and power production. According to the International Energy Agency (IEA) projections, global coal demand is expected to grow by 1.4% in 2023. Despite the continuous development of new energy technologies, coal remains a primary pillar of global electricity supply^1–3^. Taking China as an example, coal demand is forecast to increase by 3.5% in 2023, reaching 4,679 million tons, with demand in the power sector rising by 4.5% and non-power uses growing by 2%. Furthermore, China’s coal consumption is projected to rise from 87.54 exajoules in 2022 to 91.94 exajoules in 2023^4,5^. These data clearly demonstrate that coal continues to hold a significant position in current and future energy structures, providing ongoing power support for global industrial development and economic growth. However, the traditional coal combustion methods, which support this demand, are inefficient. The thermal efficiencies of these methods are only 35%–40%, far below the theoretical maximum. Approximately 2%–5% of coal dust remains as unburned carbon in fly ash, resulting in incomplete combustion^6^. Additionally, sensible heat losses from flue gases account for about 10%–15%, while chemical heat losses from unburned coal dust amount to roughly 2%–4%. Combustion instability and multi-stage energy losses lead to overall energy conversion efficiencies typically not exceeding 45%^7,8^. These inefficiencies not only contribute to significant energy waste but also indirectly increase carbon dioxide emissions, posing challenges to both the environment and economic development. Therefore, addressing the limitations of traditional coal combustion methods is essential to improving efficiency and reducing their environmental impact, ensuring coal remains a viable energy source in the future^9^.

Detonation is a combustion mode where fuel ignition propagates at supersonic speeds, typically exceeding 1000 m per second, offering higher efficiency compared to conventional combustion methods (a few meters per second)^10^. Based on the Zeldovich–von Neumann–Döring (ZND) theory, the detonation process involves shock compression, a chemical reaction zone, and heat release. It utilizes high-pressure, high-temperature shock waves to instantly ignite fuel, rapidly converting chemical energy into thermal and mechanical energy. This combustion method can reduce unburned carbon emissions to 1%-3%, significantly enhancing combustion efficiency^11^. Detonation engines are primarily categorized into Pulse Detonation Engines (PDE) and Rotating Detonation Engines (RDE), based on their implementation. PDEs achieve efficient fuel combustion through periodically generated detonation waves. They have demonstrated notable superiority in coal powder detonation combustion, improving coal powder energy utilization and reducing environmental pollution. PDE are currently considered a potentially mature solution for high-efficiency coal utilization. In recent years, notable progress has been made in both domestic and international research utilizing PDE for coal dust detonation^12,13^. The primary research approaches have focused on experimental studies and numerical simulations.

In experimental research, investigators employ specially designed and constructed PDE or PDE-like apparatuses to examine the detonation combustion characteristics of coal dust under varying conditions. These studies primarily focus on the effects of coal particle size, concentration, pressure, temperature, and oxidizer type on detonation combustion properties. Blunck et al.^14^ from Oregon State University designed a powder fluidization apparatus and employed high-speed photography to ensure uniform powder fluidization. Detonation experiments using methane/coal/oxygen mixtures were conducted in a pulse detonation engine (PDE). However, experimental results indicated that coal powder did not significantly enhance gas detonation. The researchers posited that the coal particles’ diameter exceeding 10 mm in the study may have impeded rapid reaction and sustained detonation wave propagation. Vasilev et al.^15^ and Pinaev et al.^16–18^, combining theoretical derivations and experimental data from their self-developed PDE apparatus, discovered that by setting different fuel equivalence ratios and experimental forces, under certain conditions, coal dust exhibited a significant enhancement in detonation pressure after the introduction of methane/oxygen gas. This indicates that methane can serve as a detonation gas in coal dust detonation. Pinaev investigated the characteristics of detonation waves in methane, coal dust, and oxygen mixtures. He utilized two pre-detonation tubes, each 0.9 m long with an internal diameter of 50 mm. The initiation energy density reached 11.5 MJ/m^2^, with combustion product temperatures of 4000 K. When applied to a 3-m diameter coal mine tunnel, this energy density would result in an initiation energy of 81.39 MJ, equivalent to 19.4 kg of TNT. The ignited mixture rapidly achieved stable detonation wave velocities within 75 cm of the initiation point. It is noteworthy that obtaining uniform dust cloud concentrations in experiments presents a significant challenge. Excessive concentration gradients may lead to detonation extinction after propagating a certain distance. Consequently, experiments sometimes require more powder than theoretically calculated. Ni et al.^19^ investigated the influence of coal powder morphology on detonation. They discovered that the shape of coal particles plays a crucial role in the detonation process. The addition of porous anthracite enhanced detonation intensity, whereas flaky anthracite weakened it. Pinku et al.^20–22^ research focuses on performance optimization of Pulse Detonation Combustors (PDC), particularly in the application of pulverized coal detonation combustion technology. Their research demonstrates that the curvature radius of U-shaped channels significantly influences the propagation of detonation waves in hydrogen-air mixtures, with a U-bend radius of 3.5 cm accelerating the transition from deflagration to detonation and improving overall combustion efficiency. Additionally, they studied the effects of shrouded injectors on initial vortex formation and combustion efficiency under various fuel conditions, finding that shrouded injectors effectively enhance the formation of initial vortices, thereby improving the overall performance and efficiency of the combustor.

Numerical simulation techniques have played a crucial role in researching coal dust detonation and combustion. Researchers employ computational fluid dynamics (CFD) and reaction kinetics models to conduct detailed simulations of coal dust detonation and combustion processes. These simulations enable a profound understanding of complex mechanisms such as chemical reactions, heat transfer, and fluid dynamics during combustion, thereby facilitating the optimization of combustion conditions and parameters. Salvadori et al.^23^ considered coal particles as a gas phase and employed the Euler-Euler approach for simulation. Zhu et al.^24,25^ examined the coal devolatilization process during thermal decomposition. For cases where the particle volume fraction is significantly less than 10%, they employed an Euler-Lagrangian approach for simulation. The numerical results from both methods indicate that the introduction of coal particles into the detonation field does not produce a notable effect, with the detonation primarily sustained by gaseous fuel reactions. Simulation results by Zhu et al.^26^ indicate that carbon powder can react with air to sustain stable detonation during explosive combustion. Dunn et al.^27^ conducted one-dimensional simulations of carbon/hydrogen/air detonations for 28 conditions with varying particle sizes and equivalence ratios. Results showed that the von Neumann pressure, von Neumann temperature, final temperature, and final pressure were all higher than in pure gas-phase detonations, demonstrating that carbon participates in and enhances the detonation process. Zhang et al.^28^ conducted one-dimensional simulations of carbon/carbon monoxide/hydrogen/air detonations and discovered that small particles significantly enhanced the detonation process. Shi et al.^29^ conducted a detailed investigation into the structure of methane/coal/oxygen mixture detonations. The findings reveal that particulate characteristics significantly influence the detonation structure. Enhanced carbon combustion, induced by unburned gas pockets, results in elevated localized heat release. This phenomenon concurrently promotes the formation of compression waves, thereby intensifying the leading shock wave and yielding higher combustion efficiency. The aforementioned numerical simulation results indicate that establishing appropriate detonation reaction mechanisms and numerical models is a key focus in the study of gas–solid two-phase pulse detonation. It is also crucial for achieving correspondence between simulation and experimental data.

The above research shows that pulverized coal is feasible as a detonation fuel, and its low cost and high safety characteristics make its application in PDE worthy of expectation, but there are also some problems, including: the complexity of coal powder; instability of the combustion process; complexity of PDE systems. Currently, the characteristics of detonation waves in different types of coal are not fully understood. For practical engineering applications of PDE, it is crucial to comprehend the propagation features of detonation waves in various coal types, such as wave stability and detonation combustion efficiency. Bykovskii and collaborators^30–34^ pioneered the study of detonation combustion in various coal types, including anthracite and lignite. Using a custom-designed PDE-like apparatus, they conducted detonation experiments on different coal powders, observing variations in detonation pressure waves and stability within the detonation flow field. Xu^35^ and Roy^36^ observed similar phenomena in experiments and computer simulations, respectively, indicating that coal powder detonation waves exhibit different combustion modes and efficiencies under varying conditions. Combustion efficiency is a crucial factor affecting PDE operation, with higher efficiency leading to improved energy utilization of coal. However, few researchers have focused on detailed studies of combustion efficiency for different coal types in PDEs, particularly in the context of emerging deep coal fluidized mining technologies.

To further address the aforementioned issues, with the research background of fluidized mining of deep coal resources^37^, a series of PDE experiments were conducted on coal powder/methane/oxygen mixtures at room temperature. These experiments revealed the detonation evolution process of different coal types under varying equivalence ratio conditions in PDE. Concurrently, numerical simulations were performed based on the PDE model to supplement key experimental parameters. By combining the numerical simulation data with experimentally measured data, the combustion efficiency of different coal types was determined. This research provides valuable scientific insights for further understanding coal powder detonation phenomena and improving combustion efficiency in PDE devices.

## Materials and methods

### Materials

Analysis was performed on four distinct pulverized coal types sourced from the Tashan Coal Preparation Plant in Shanxi, China. The assessment adhered to the GB/T-30732-2014 standard to evaluate the industrial quality characteristics of these specimens. Table 1 presents the findings in detail, showing a trend where ash and volatile content rise as the coal’s rank descends from anthracite to peat, whereas fixed carbon and calorific value drop. For the experiments, the coal samples were finely ground to under 10 μm using a high-energy planetary ball mill, with a minimum of three repetitions undertaken. The particle size distribution of pulverized coal was measured using a laser particle size analyzer (Malvern Mastersizer 3000). The results showed that the particle size distribution of the pulverized coal samples ranged from 1 μm to 10 μm, with a D50 value of approximately 5.265 μm. This particle size range was chosen to ensure sufficient surface area for combustion reactions and to enhance the detonation process. The experiment provided an explanation for the ash content of coal powder that does not undergo a reaction. Under oxygen-enriched conditions, with a fixed equivalence ratio of 1.8, the complete detonation state of the coal powder fuel was investigated. At different coal powder mass flow rates, the corresponding oxygen mass flow rates were adjusted proportionally to maintain the equivalence ratio. As shown in Table 2, the oxygen mass flow rate increased with the coal powder mass flow rate, ensuring consistency in the fuel-oxidizer mixing conditions. Oxygen-enriched conditions (with an oxygen purity of 99.995%) were selected to enhance combustion stability and detonation efficiency.

**Table 1:** Results of industrial analysis for coal samples (air dried basis).

Sample	M_ad_/%	A_ad_/%	V_ad_/%	FC_ad_/%	LHV/(MJ/kg)	D50
Anthracite	1.15	6.79	14.61	77.45	31.87	5.16
Bituminous coal	1.28	15.24	15.84	67.64	25.98	5.61
Lignite	1.33	25.81	34.69	38.17	21.15	5.12
Peat	1.24	36.01	33.94	28.81	16.62	5.17

Mad, Moisture content on air-dried basis; Aad, Ash content on air-dried basis; Vad, Volatile matter content on air-dried basis; FCad, Fixed carbon content on air-dried basis; LHV, Lower Heating Value.

**Table 2:** Experimental design operating conditions.

No	Coal/(g/s)	O_2_/(g/s)	CH_4_/(g/s)	CH_4_/%	Equivalence ratio (c)
1	30 ± 0.2	51.78 ± 0.35	3.99 ± 0.03	13.35 ± 0.09	1.8 ± 0.02
2	60 ± 0.25	103.56 ± 0.43	7.98 ± 0.03	13.35 ± 0.05	1.8 ± 0.01
3	90 ± 0.4	155.34 ± 0.69	11.97 ± 0.05	13.35 ± 0.06	1.8 ± 0.01
4	120 ± 0.5	207.12 ± 0.86	15.96 ± 0.07	13.35 ± 0.05	1.8 ± 0.01

### Experimental test set up

**Fig. 1:**
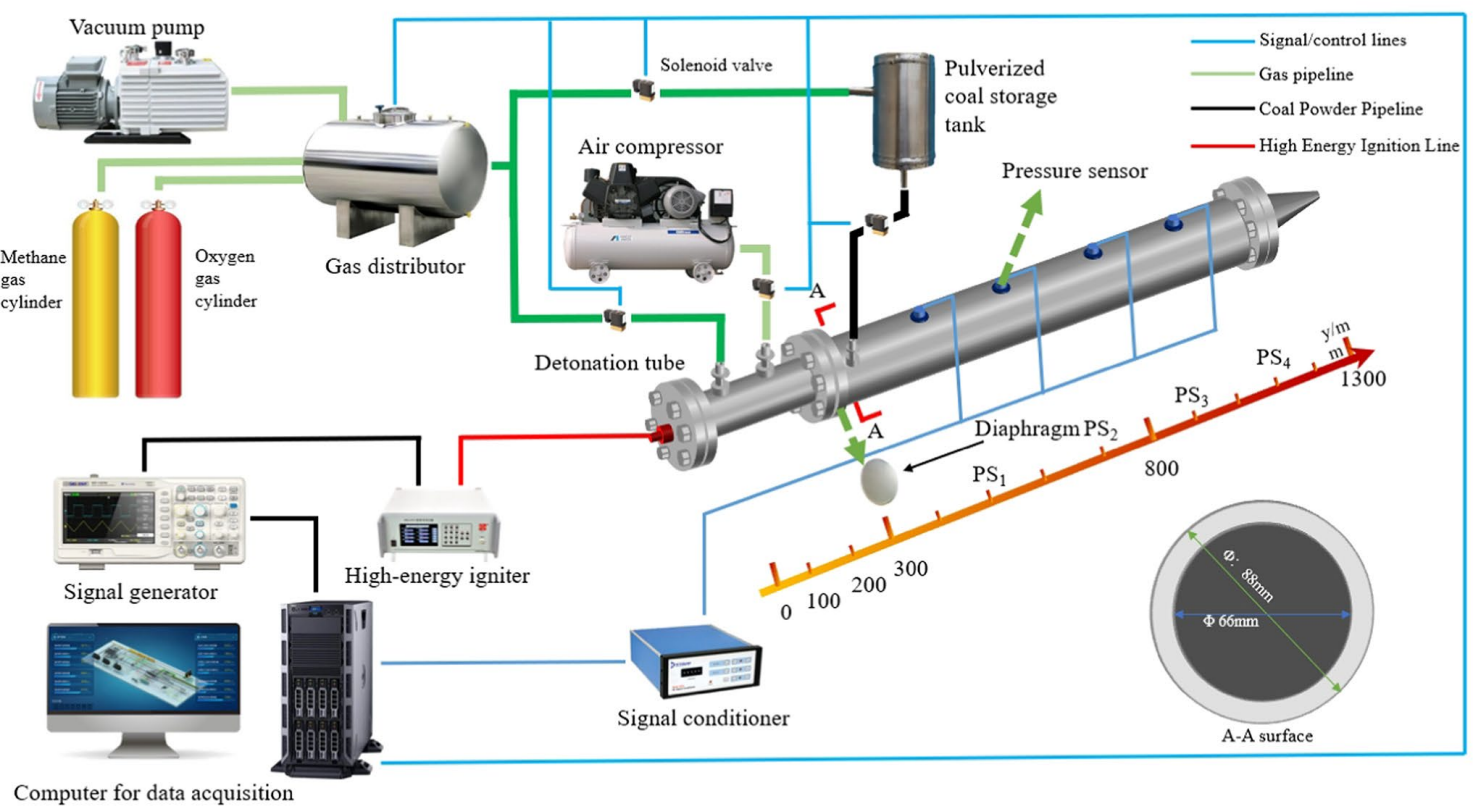
Detonation test equipment^35^.

Figure 1 illustrates the layout of the experimental setup, which includes a main combustion chamber customized for the Pulse Detonation Engine (PDE) with improvements based on previous research^38,39^. The interior of the combustion chamber is an empty cavity with dimensions as shown in Fig. 1. This PDE also encompasses systems for fuel and oxidizer supply, ignition, as well as control and data acquisition components. The pulse detonation chamber (PDC) is made up of a fuel chamber, a methane inlet, and a powder inlet. As depicted in Fig. 3, the PDC combustion chamber has a coaxial annular construction with an outer diameter of 100 mm, an inner diameter of 66 mm, and a length of 1000 mm. In operation, fuel and oxygen are pre-injected into this chamber and then conveyed into the mixing zone through evenly spaced apertures. The coal powder delivery system mainly includes a methane pipeline, a powder storage chamber, a piston mechanism, and a stepper motor. During fueling, ultrafine coal powder is introduced into the storage chamber, compacted with the piston mechanism and stepper motor, then delivered at a controlled flow rate with methane to carry it into the PDC for injection. To ensure uniform injection and efficient combustion of pulverized coal, the key parameters of the injection system were optimized. The nozzle diameter for the injection of the pulverized coal and methane mixture is 5 mm. This diameter was selected to effectively disperse the pulverized coal while preventing nozzle clogging. The total pressure drop from the pulverized coal storage chamber to the PDC injection port is approximately 0.15 MPa, achieved by controlling the pressing force of the piston mechanism and adjusting the methane delivery pressure to ensure a stable pulverized coal feed rate. Through steady-state control of the stepper motor, the feed rate of pulverized coal can be adjusted within the range of 20 g/s to 155 g/s, enabling the study of the effects of different pulverized coal flow rates on detonation characteristics. This ensures proper mixing and uniform distribution, promoting efficient combustion. Pipelines leading to the PDC and fluidized bed are equipped with sonic nozzles to stabilize the methane flow rate. The setup necessitated adjusting upstream pressure in the sonic nozzles on each pipeline. An Endress Hauser mass flow meter (Pro-mass F 200) provides measurements of the oxygen mass flow rate, with a maximum error margin of ± 0.2%. When the PDC is in operation, four high-frequency pressure sensors (PS_1-4_) are mounted on the detonation tube to observe the continual detonation status of lignite coal powder. These PCB-113B26 pressure sensors feature a response time under 0.6 microseconds, a 600 kHz sampling rate, sensitivity ranging from 0.6–1.6 V/MPa, and a maximum range of 6.8 MPa. To ensure the reliability of the fuel supply system, preliminary fluidization experiments were carried out to calibrate the correlation between methane flow and the coal powder mass flow rate.

### Experimental procedure

**Fig. 2:**
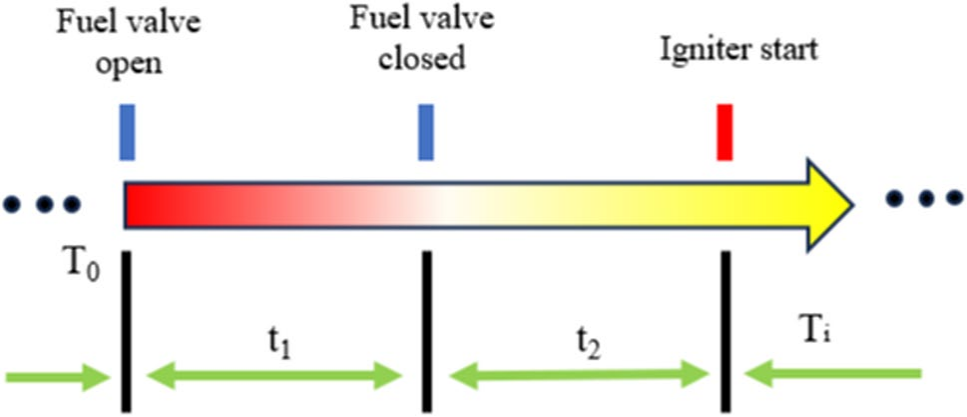
Pulse detonation chamber (PDC) operation time sequence.

Before conducting the detonation experiments, selected coal samples undergo a preparatory stage. This entails pulverizing the coal in an inert argon atmosphere using a planetary ball mill (such as the PULVERISETTE 6) to prevent oxidation and produce particles of various sizes. A homogeneous mixture of methane and oxygen is then introduced into the combustion chamber by a compressor. A pressure gauge mounted on top of the chamber provides real-time pressure data. Figure 2 depicts the ignition sequence for the detonation test, where a timing program controls solenoid valves and igniters with millisecond precision. It presents the operational time sequence of the PDC, which is crucial for achieving precise fuel and oxidizer mixing, ignition control, and the detonation process. By precisely controlling the operational timing, we can ensure the stability and repeatability of the detonation process, thereby accurately evaluating the detonation characteristics of different coal types in the PDE. This is of significant importance for achieving the study’s goal of assessing the efficiency and energy release characteristics of coal powder. Data acquisition is performed using the NI PXIe-6514 digital input module together with the PXIe-6378 multifunction acquisition card, allowing for continuous recording of pulse detonation wave propagation signals at a single-channel rate of 3.5 MS/s. The procedure starts with the fuel solenoid valve opening at time T_0_, followed by a pause interval t_2_. Then, the igniter triggers the internal fuel mixture, indicating the start of the i-th ignition cycle.

### Numerical simulation

The numerical simulation study supplements experimental results by simplifying the model to a certain extent. All simulations in this article were conducted using the commercial Computational Fluid Dynamics (CFD) software Ansys Fluent. The gradient solution is based on the least squares cell method. The flow equations are solved using a first-order upwind scheme, turbulence and specific dissipation equations use a second-order scheme, and transient equations employ a first-order implicit format. The Shear Stress Transport (SST) k-ω turbulence model is used, effectively combining the robustness and accuracy of the near-wall model. For the discrete phase, the Discrete Phase Model (DPM) was employed. In the DPM settings, set the discrete-phase interaction interval (Interaction Interval) to 1. To enhance computational efficiency, the parcel method was used within the DPM. Each parcel represents 1,000 actual particles. This value was chosen based on a balance between computational accuracy and resource requirements. On one hand, the parcel method significantly reduces the computational resources and time needed; on the other hand, by selecting an appropriate number of particles per parcel, the accuracy of the simulation results is ensured. When coal dust is injected as a discrete phase, the effects of pressure gradient force, virtual mass force, and Discrete Element Method (DEM) collisions on particles are considered, and a spherical drag law is applied to the particles. Gas chemical reactions use a laminar finite rate reaction model, and the reaction rate constants are calculated using the Arrhenius formula:

1 $$k=\text{A}{T}^{b}exp(-\frac{{E}_{a}}{RT})$$

where, *k* represents the reaction rate constant, *A* is the pre-exponential factor, *b* is the temperature exponent, *E*_*a*_ stands for the activation energy of the reaction, R represents the universal gas constant. The parameters for the chemical reaction are shown in Table 3.

**Table 3:** Chemical mechanisms (calculated based on experimental conditions).

No	Reaction	A	*E*_*a*_ (kJ/mol)
1	C_(s)_ + 0.5 O_2_ = CO	1.09e + 11	1.089e + 8
2	Volatile = 1.7060 CO + 1.543H_2_O	3.12e + 10	1.8e + 8
3	CH_4_ + 2O_2_ = CO_2_ + 2H_2_O	1.58e + 13	2.03e + 8
4	CO + 0.50_2_ = CO_2_	1.26e + 11	1.26e + 8
5	CO_2_ + H_2_ = CO + H_2_O	7.94e + 10	2.88e + 8

According to Table 3 the primary distinctions among the four coal types lie in their volatile matter and fixed carbon content. Consequently, this study’s numerical simulations will set parameters based on the volatile matter and fixed carbon content of the different coal types outlined in Table 1 to differentiate and represent these four varieties.

**Fig. 3:**
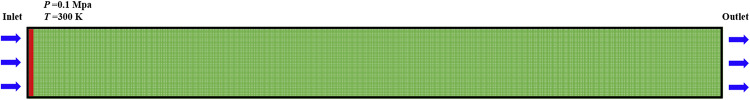
Two-dimensional model.

The combustion chamber model was set to the actual size. The two-dimensional diagram of the model is shown in Fig. 3. According to the dimensions of the main combustion chamber of the experimental PDC, the length of the combustion chamber is 1000 mm and the inner diameter is 66 mm. Consistent with the experimental conditions, the fuel inlet boundary is set as the mass inflow port, and different pulse detonation results can be obtained by setting different fuel flow rates. Pulverized coal, as a discrete-phase particle, is injected into the pulse detonation engine (PDE) through a dedicated inlet. Specifically, the pulverized coal enters the detonation tube independently via a specialized coal inlet, while methane serves as a carrier gas and mixes with the coal particles. The oxidizer is injected separately through another inlet. After the pulverized coal, methane, and oxidizer are thoroughly mixed in the mixing zone within the detonation tube, a detonation combustion reaction occurs. In numerical simulations, the initial temperature of the pulverized coal is set to 300 K, corresponding to room temperature conditions. This setting aligns with actual experimental conditions, as no preheating treatment is applied to the pulverized coal. The outlet is set as a pressure outlet boundary with an ambient pressure of 0.1 MPa and a temperature of 300 K (Synchronize with the actual experimental environment). The boundary condition for the Discrete Phase Model (DPM) is set to escape.

**Fig. 4:**
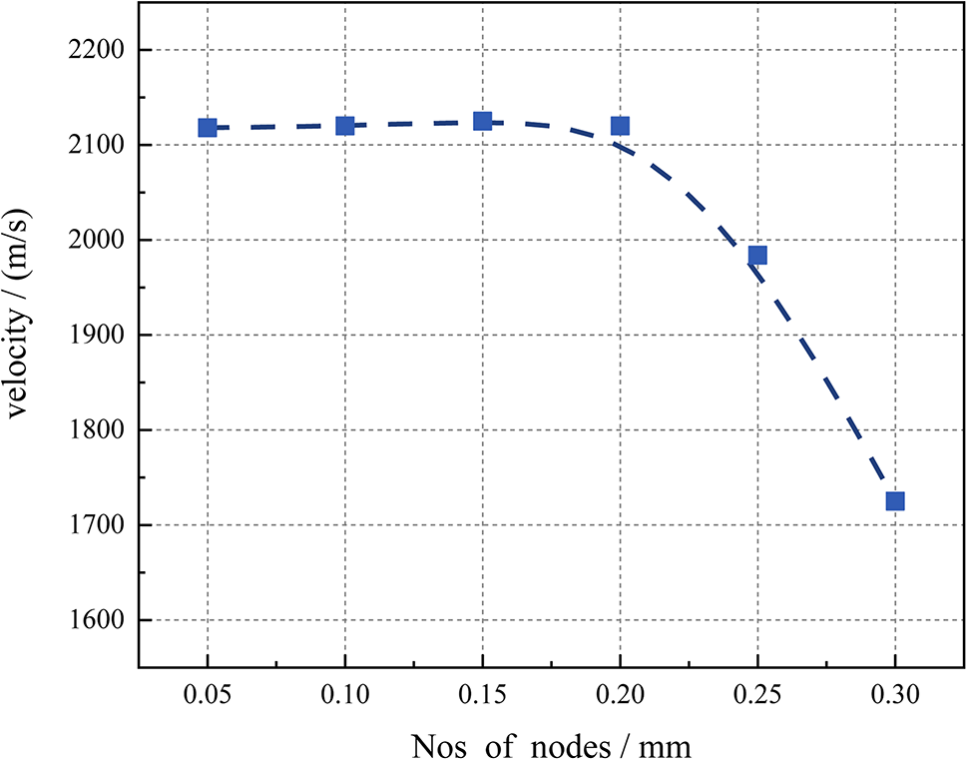
Grid independence test of compzutational domain.

Accurate results are influenced by the resolution of the grid. The grid can be refined to a certain extent; beyond this point, further refinement has a negligible impact on the results. This phenomenon is called Grid Independence Limit (GIL). The velocity of the detonation wave in the pulse combustion chamber is used as a parameter to test the grid independence of the computational domain. Figure 4 shows the different levels of grid refinement used for the grid independence test. The results indicate that for cell sizes ranging from 0.05 to 0.20 mm, the detonation wave speed remains essentially consistent. When the cell size exceeds 0.2 mm, the detonation wave speed begins to decrease. Considering computational costs, a grid size of 0.2 mm was chosen for subsequent simulation experiments.

The control equations used to describe the pulse detonation flow field are the Navier–Stokes (N–S) equations and the species transport equations, as follows:

2 $$P=\rho RT$$

3 $$E=h-\frac{P}{\rho }+\frac{{U}^{2}}{2}$$

4 $$\tau = \mu [\nabla U + \left( {\nabla U} \right)^{T} - \frac{2}{3}(\nabla \cdot U)I]$$

5 $${S}_{mass}=\frac{\partial \rho }{\partial t}+\nabla \cdot (\rho U)$$

6 $${S}_{mom}=\frac{\partial (\rho U)}{\partial t}+\nabla \cdot \left(\rho UU\right)+\nabla P-\nabla \cdot \tau$$

7 $${S}_{species,m}+{\dot{\omega }}_{m}=\frac{\partial (\rho {Y}_{m})}{\partial t}+\nabla \cdot \left[U({\rho Y}_{m})\right]+\nabla \cdot {s}_{m}$$

8 $${S}_{energy}+{\dot{\omega }}_{T}=\frac{\partial (\rho E)}{\partial t}+\nabla \cdot \left[\left(\rho E+P\right)U\right]+\nabla \cdot (\tau \cdot U)$$

where, $U$ represents the velocity vector; ${Y}_{m}$ and ${s}_{m}$ respectively denote the mass fraction and diffusion flux of component m; $\mu$ represents dynamic viscosity; $I$ denotes the identity tensor; $h$ is the total enthalpy of the reactants. $m$ represents the total number of components; $\nabla$ is the Hamiltonian operator; ${\dot{\omega }}_{T}$ denotes the rate of chemical reaction; ${\dot{\omega }}_{m}$ is the rate at which component m is produced or consumed by chemical reactions; $R$ stands for the gas constant; ${S}_{mass}$, ${S}_{mom}$, ${S}_{energy}$, and ${S}_{species,m}$ respectively represent the mass source term, momentum source term, volumetric heat source term, and the source term produced by component m. $\tau$ and $E$ represent the viscous stress tensor and total energy, respectively.

**Fig. 5:**
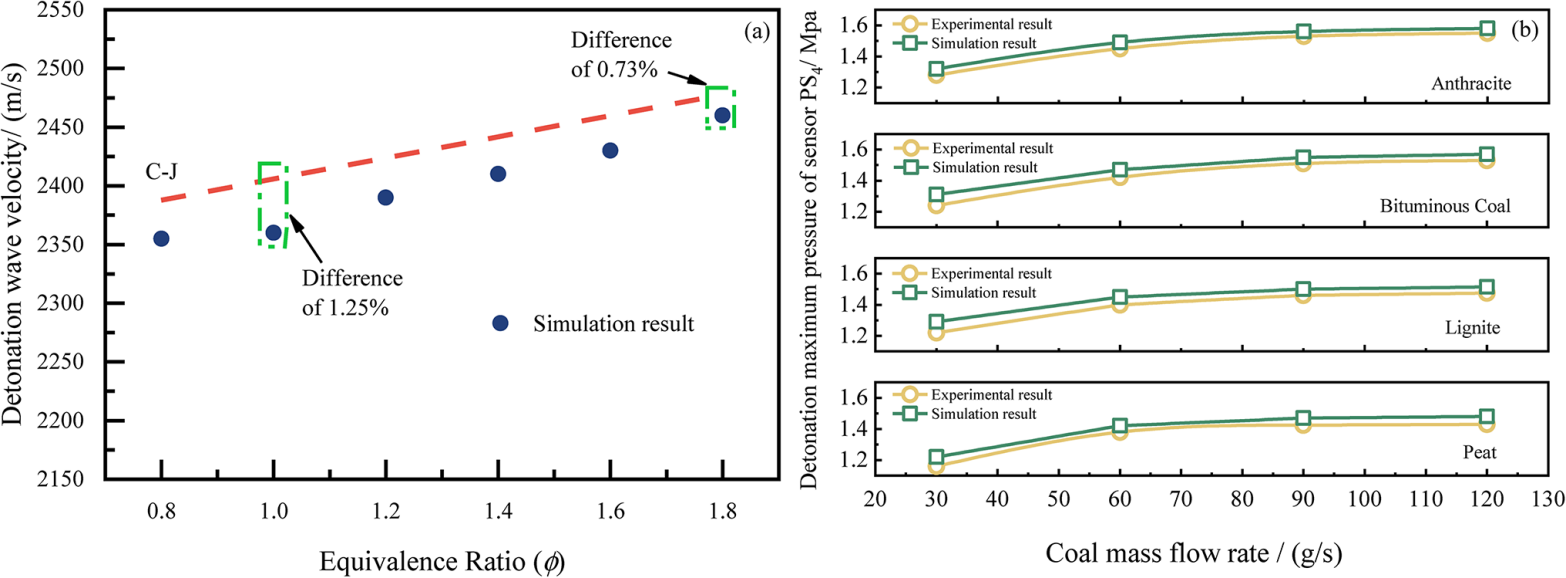
Simulation results verification. (**a**) Comparison with theoretical C-J velocity; (**b**) Comparison with experimental result).

The verification results of this solver are illustrated in Fig. 5. Firstly, Fig. 5a compares the detonation wave speeds obtained by the solver with the theoretical detonation wave speeds calculated using NASA’s CEA software. As the equivalence ratio increases from 0.8 to 1.8, both sets of detonation wave speeds exhibit a consistent trend, with a minimum error of 0.73%. Subsequently, Fig. 5b presents a comparison between the solver’s simulation results and experimental results. It indicates that under varying coal powder conditions, the numerical simulations and experimental outcomes show a consistent trend as the mass flow rate of coal powder increases.

## Results

### PDE pressure characteristics of pulverized coal

#### Typical characteristics of single PDE pressure of pulverized coal

The primary distinction between detonation and deflagration lies in the completeness of combustion and the intensity of released energy. Detonation releases energy more forcefully by propagating pressure waves outward, resulting in a more intense detonation pressure wave. During experimental observations, numerous instances of deflagration were noted, characterized by significantly lower pressures under identical conditions. Therefore, comparing the pressure characteristics of these two combustion states is essential.

**Fig. 6:**
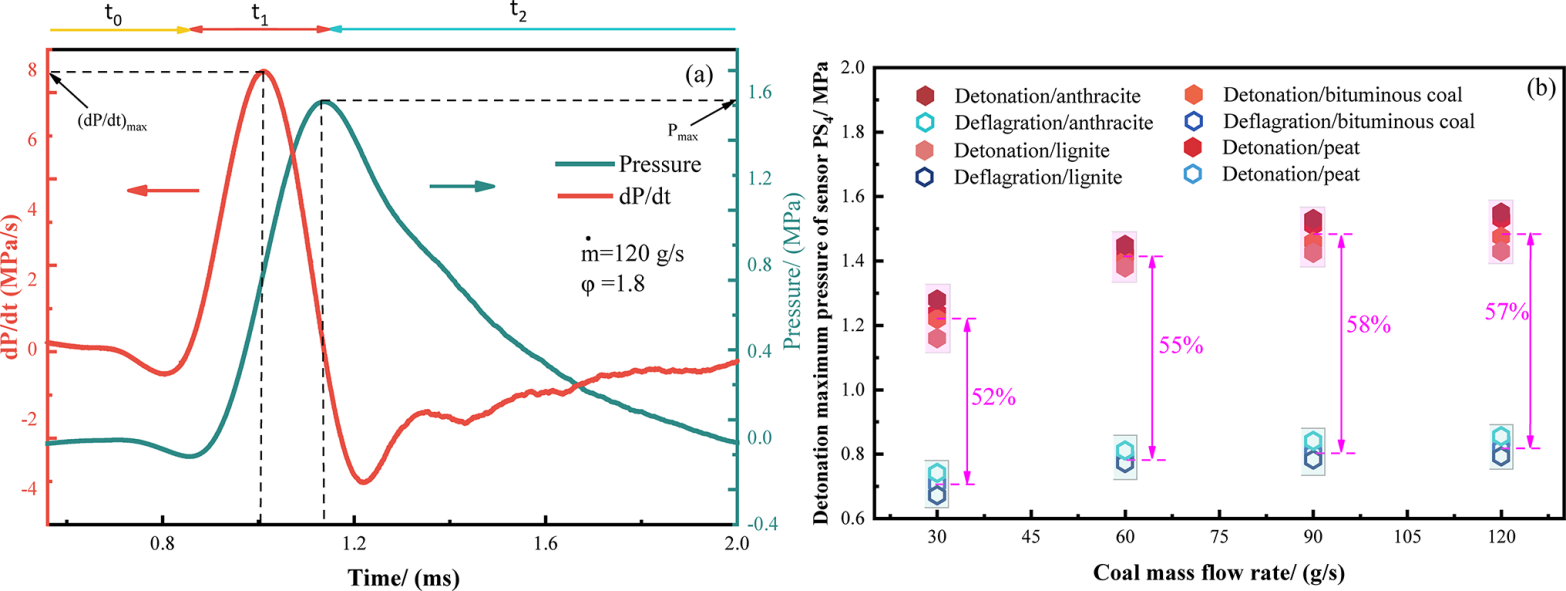
The typical pressure characteristic curve of PDE detonation wave is measured by PS_4_. (**a**) Typical pressure characteristics of monopulse detonation wave; (**b**) Comparison of two modes of detonation pressure wave).

Figure 6 illustrates the pressure characteristics as monitored by the PS_4_ pressure sensor for both modes. Figure 6a depicts the characteristic pressure curve of a typical single-pulse detonation, which can be divided into three stages: the pre-detonation steady-state pressure stage (t_1_), the rapid pressure rise stage (t_2_), and the gradual pressure decline stage (t_3_). The primary energy release occurs during t_2_, reaching the maximum pressure (*P*_max_). By differentiating with respect to time, the maximum pressure rise rate (dP/dt)_max_ during t_1_ can be calculated, thus the detonation wave velocity can be calculated^34^. Figure 6b compares the maximum pressure characteristics of detonation and deflagration. The pressure trends for the four types of coal (anthracite, bituminous coal, lignite, and peat) are generally consistent. As the mass flow rate of coal dust increases (from 30 to 120 g/s), the *P*_max_ for both detonation and deflagration gradually rises. The P_max_ for detonation and deflagration differs by approximately 50–60%, aligning with the conclusions of reference^35^ regarding detonation states.

#### PDE continuous operating pressure characteristics

The previous section discussed the variations in *P*_max_ under two modes based on a single pressure sensor (PS_4_). However, the dynamic nature of pulse detonation waves necessitates a more comprehensive analysis. Examining the pressure change characteristics of the sensor over time can give a deeper understanding of the whole pulse dynamic process.

**Fig. 7:**
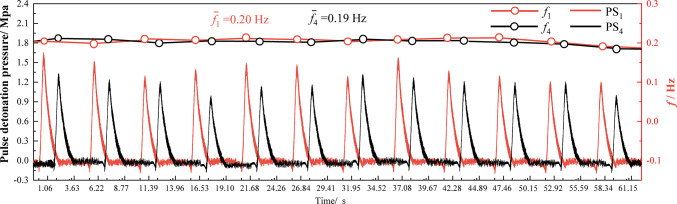
Dynamic pressure characteristics measured by PS_1_ and PS_4_ (lignite: 90 g/s).

Figure 7 shows the dynamic typical pressure characteristic curve of brown coal under the condition of 90 g/s continuous 12 times pulse detonation. The pressure signals of PS_1_ and PS_4_ appear alternately, with the peak pressure of PS_1_ occurring before that of PS_4_, demonstrating the unidirectionality of the pulse detonation wave. The frequency in the figure represents the reciprocal of the time interval between consecutive periodic pulse pressure peaks (Including single pulse detonation pressure time and reset time). It is evident that the frequency at the two sensor monitoring points is approximately 0.2 Hz, maintaining a consistent stability.

9 $${v}_{p}=\frac{{l}_{i+1}-{l}_{i}}{\Delta t}$$

where, ${v}_{p}$ represents the detonation wave velocity between adjacent monitoring points, ${l}_{i+1}$ and ${l}_{i}$ are the distances between two consecutive monitoring points, set to 200 mm in this study, $\Delta t$ is the time difference between successive pulse monitoring events. Data monitored by PS_1_-PS_4_ sensors during working hours can be used to analyze the pressure distribution of detonation pressure waves.

**Fig. 8:**
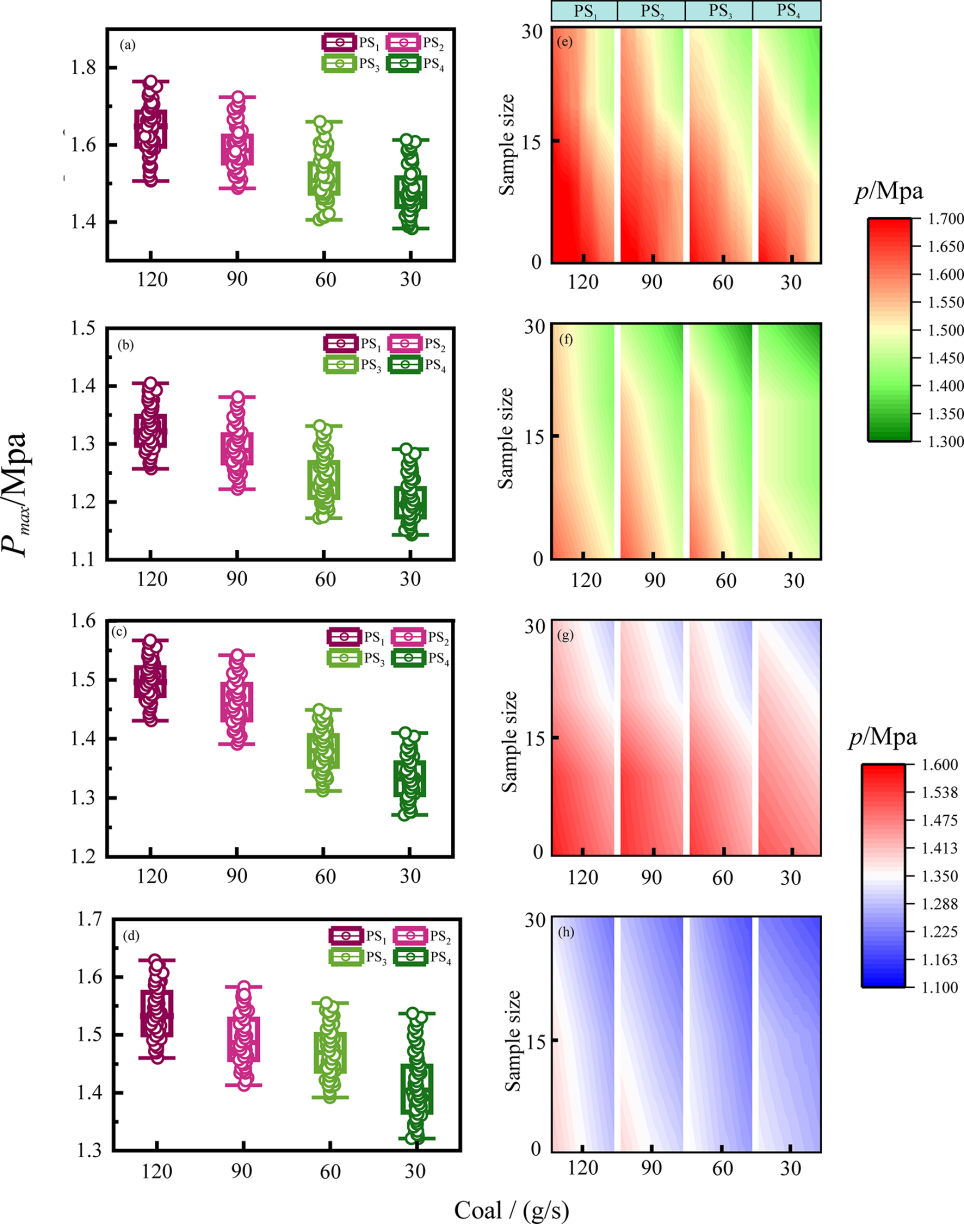
Pulse detonation pressure data of four types of coal at different flow rates measured by sensor PS_1–4_. (**a–d**) Detonation data distribution of anthracite, bituminous coal, lignite and peat; (**e**–**h**) distribution cloud map of detonation data points of anthracite, bituminous coal, lignite and peat).

As shown in Fig. 8, the detonation pulse pressure data for four types of coal powder (Anthracite, Bituminous coal, Lignite, and Peat) under different operating conditions from Table 2 is presented. Under the same coal powder flow conditions, the pressure at PS_1_ is the highest, indicating that the detonation wave consistently propagates from the head to the tail of the pulse tube. Although the detonation pressure wave always propagates unidirectionally, the pressure propagation characteristics vary among different coal powders. This is clearly evident from Fig. 8a–d, where the pressure trends at PS_1_-PS_4_ differ. The trend can be expressed by the slope of the pressure change (as shown in Eq. 8, enabling the calculation of detonation wave velocity. It can be deduced that under the same flow conditions, different types of coal will impact the detonation wave speed. Figure 8e–h show the detonation wave data from 30 heap test stack clouds for different coal powders. It granulates the impact of coal powder flow on the detonation waves in detail. Under the operating conditions in Table 2, a flow rate of 120 g/s produced the strongest detonation pressure.

### Efficiency analysis of pulverized coal detonation combustion

#### Quantitative calibration of P–V relationship of pulverized coal detonation

The detonation combustion efficiency (DCE) refers to the ratio of the actual heat generated in the detonation combustion chamber to the total theoretical heat that can be released when the fuel is completely combusted. This ratio reflects the extent and intensity of the chemical reactions occurring within the detonation combustion chamber. Higher chemical reaction intensity leads to greater fuel consumption rate and heat release rate, resulting in improved combustion efficiency. DCE is defined in Eq. (8):

10 $${\eta }_{DCE}=\frac{\Delta {T}_{i}}{\Delta {T}_{th(i)}}=\frac{{\overline{T}}_{i}-{\overline{T}}_{0}}{{\overline{T}}_{th(i)}-{\overline{T}}_{0}}$$

11 $${T}_{i}=\frac{{P}_{i}V}{nR}={K}_{s}{P}_{i}$$

12 $$T_{{th\left( i \right)}} = \frac{{\Delta LHV_{{coal}} m_{f} + m_{a} C_{{ga}} \bar{T}_{i} ^{*} + m_{f} C_{c} \bar{T}_{i} ^{*} }}{{C_{{pg}} (m_{a} + m_{f} )}}$$

where, ${\eta }_{DEC}$ represents detonation combustion efficiency; ${T}_{i}$ is the temperature value at position i, ${P}_{i}$ refers to the pressure value at position i, ${K}_{s}$ is the *P*–*V* characteristic coefficient, ${\Delta LHV}_{coal}$ is the lower heating value of coal before and after detonation, ${C}_{ga}$ is the specific heat at constant pressure for gaseous fuel, ${C}_{c}$ is the specific heat at constant pressure for coal, ${C}_{pg}$ is the specific heat at constant pressure for gases generated during detonation. From Eq. 9, it is evident that the detonation pressure is directly proportional to the local temperature. During actual experiments, the transient temperature cannot be measured using sensors; hence, a numerical model is utilized to address this issue.

**Fig. 9:**
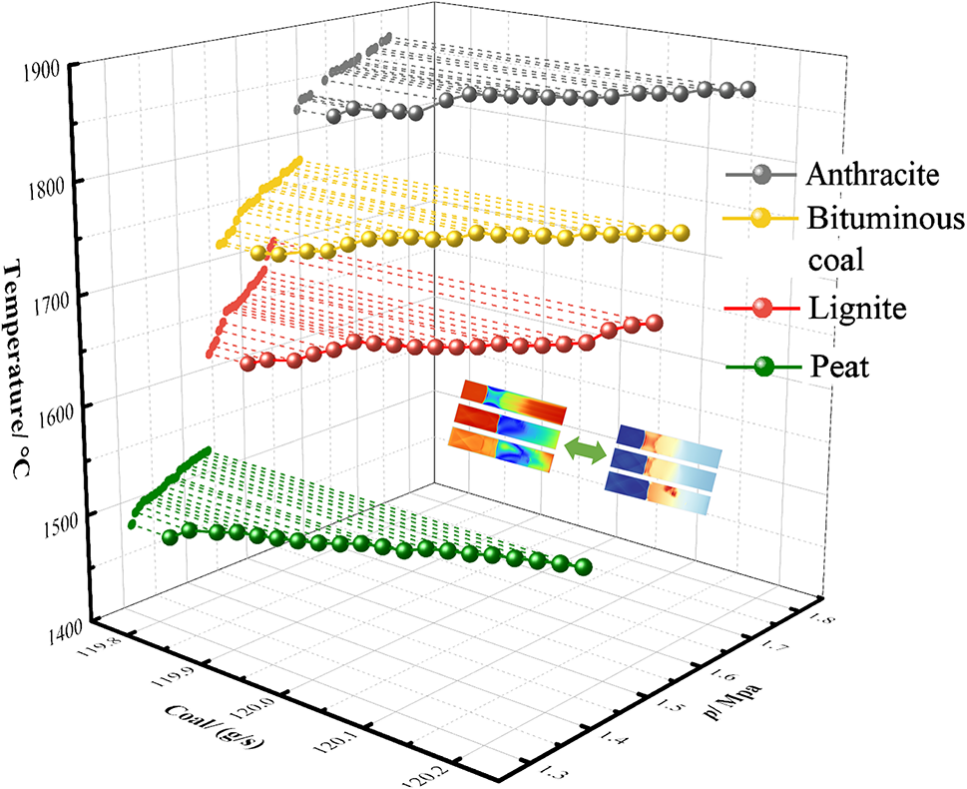
Pulverized coal/flow rate pulse detonation pressure is temperature relation.

Figure 9 shows the maximum pressure and simulation temperature results of four kinds of pulverized coal pulsed detonation at ps1 under the condition of 120 g/s. In terms of maximum pressure, the maximum pressure of anthracite coal increased from 1.599 MPa to 1.764 MPa, resulting in an increase of approximately 10.32%; bituminous coal increased from 1.491 MPa to 1.62 MPa, with an increase of about 8.66%; lignite increased from 1.463 MPa to 1.567 MPa, showing an increase of approximately 7.11%; and subbituminous coal increased from 1.291 MPa to 1.405 MPa, with an increase of around 8.84%. A comparison across the board reveals that anthracite coal had the greatest increase, with a difference of approximately 45% between anthracite coal (10.32%) and lignite (7.11%), followed by subbituminous coal in pressure changes. In terms of maximum temperature, the maximum temperature of anthracite coal increased from 1548.55 °C to 1600.55 °C, resulting in an increase of about 2.85%; bituminous coal increased from 1415.15 °C to 1476.55 °C, showing an increase of approximately 3.63%; lignite increased from 1334.65 °C to 1425.05 °C, with an increase of around 5.62%; and subbituminous coal increased from 1170.85 °C to 1215.25 °C, revealing an increase of about 3.07%. A horizontal comparison indicates that although anthracite coal had a higher initial temperature value, it had the lowest increase. Lignite exhibited the greatest increase, with a difference of approximately 83% between lignite (5.62%) and subbituminous coal (3.07%). Through a comparison of experimental data and simulation data, it can be observed that the changes in flow rate and pressure exhibit a high degree of consistency, thus validating the reasonableness of the maximum temperature simulation results. This method effectively resolves the challenge of directly measuring temperature in experiments, providing a reliable reference basis for the study of coal dust detonation characteristics.

**Fig. 10:**
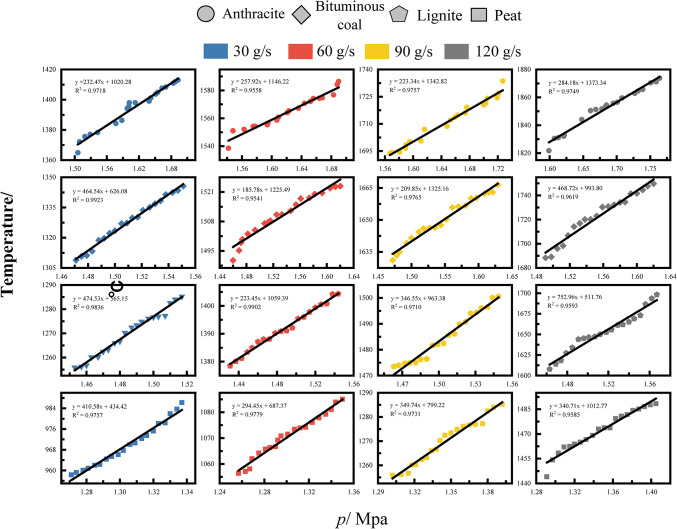
Pulverized coal/flow rate pulse detonation pressure is temperature relation.

To further validate the relationship between maximum detonation pressure and maximum temperature, Fig. 10 shows the P–V relationship under different experimental conditions. It can be seen that all coal powders exhibit linear relationships between temperature and pressure under different flow rates. The slopes and intercepts of the fitted equations for different coal powders slightly vary, indicating differences in the relationship between temperature and pressure for different coal powders. Based on the derivation of the thermodynamic state formula (Eq. 9) mentioned earlier, it is possible that there was a linear relationship between the macroscopic state parameters (P–T) before. This reflects the conservation principle of energy according to the first law of thermodynamics; in addition, detonations fundamentally belong to chemical reactions and should follow the Arrhenius formula, where the reaction rate constant is an exponential function of temperature. Therefore, an increase in pressure will accelerate the combustion reaction rate, leading to a rapid increase in temperature. The positive correlation between this reaction rate and pressure is mathematically represented by a linear relationship; meanwhile, during the combustion of coal powder, a large amount of gas (such as CO_2_, H_2_O, CO) is generated. These generated gases further increase the system’s pressure due to expansion in a high-pressure environment. Additionally, the rate of gas generation is linearly related to temperature: higher temperatures accelerate the combustion reaction of coal powder, producing more highly reactive radicals and generating more gas. The rapid diffusion and expansion of these gases directly result in an increase in pressure; utilizing the classic ZND theory of detonations, pressure increase affects the thickness of the combustion reaction zone during the detonation process, making the combustion reaction more concentrated. The reduction in the thickness of the reaction zone is conducive to achieving a high temperature state more quickly, and this compression effect of pressure on the combustion reaction zone is one of the important physical mechanisms for the establishment of the linear relationship. In conclusion, the reason why different coal powders exhibit a linear relationship between *P*_max_ and highest temperature *T*_max_ during the pulse detonation process is the result of multiple factors such as chemical reaction kinetics, gas generation and diffusion, thermodynamic equilibrium and energy conservation, physical and chemical properties of coal powder, compression effect of the combustion reaction zone, and turbulent convective heat transfer mechanism affecting and interacting with each other. These factors collectively lead to independent experimental data showing a certain macroscopic linear relationship overall, providing a theoretical basis and experimental evidence for a deeper understanding of the combustion and detonation characteristics of coal powder.

#### Quantitative analysis of pulverized coal detonation efficiency

**Fig. 11:**
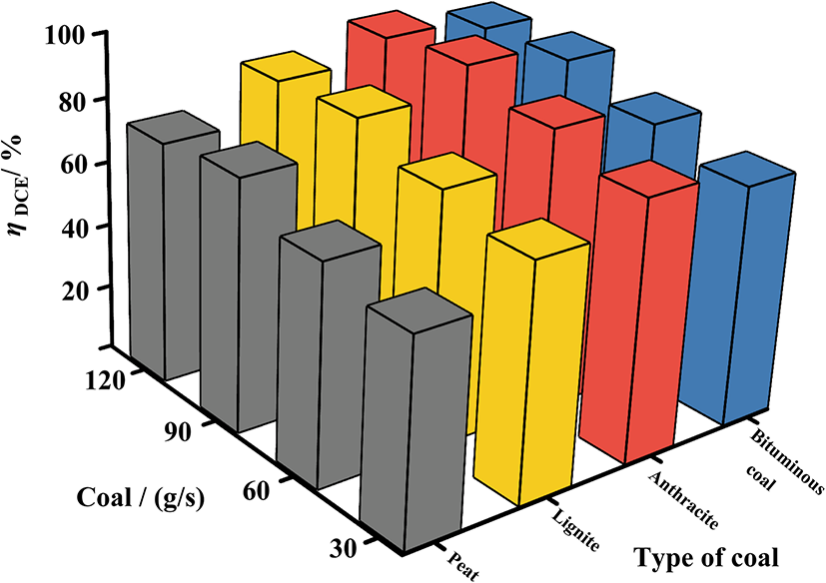
3D histogram of ${\eta }_{DCE}$ for different coal species/flow rates.

Figure 11 presents histograms showing the distribution of ${\eta }_{DCE}$ under different coal powder types and various coal powder flow rates. From the figure, it can be observed that as the mass flow rate of coal powder increases, ${\eta }_{DCE}$ exhibits an upward trend. For peat, when the mass flow rate of coal powder increased from 30 g/s to 120 g/s, the ${\eta }_{DCE}$ e rate rose from 63.1% to 75.4%, displaying a gradual upward trend. In the case of lignite, ${\eta }_{DCE}$ increased from 73.4% to 86.2%, demonstrating a clear upward trend. Under anthracite conditions, ${\eta }_{DCE}$ increased from 81.5% to 92%, indicating the most significant enhancement in combustion efficiency. For bituminous coal, ${\eta }_{DCE}$ increased from 75.7% to 88.3%, similarly rising with the increase in coal powder flow rate. The specific statistical results indicate that the ${\eta }_{DCE}$ of different types of coal powder is primarily affected by the coal powder flow rate. Although ${\eta }_{DCE}$ varies with changes in coal powder type, the impact of coal powder flow rate on ${\eta }_{DCE}$ is more pronounced. Anthracite exhibits the highest combustion efficiency under pulse detonation conditions, whereas peat exhibits relatively low combustion efficiency.

Combustion efficiency differences among coal types arise from coal quality, chemical kinetics, and physical properties. Anthracite’s high fixed carbon (77.45%) and low ash content (6.79%) promote complete combustion and higher efficiency. Peat, with low fixed carbon (28.81%) and high ash (36.01%), releases less heat, resulting in lower efficiency. High volatile matter in lignite and peat aids ignition but can lead to incomplete combustion when ash is high. Anthracite’s dense structure and high carbon content lead to higher activation energy and reaction rates, causing intense combustion under high temperatures and pressures. Lower-rank coals like peat have more oxygen-containing groups, releasing less heat and more unburned carbon. Physical properties like particle morphology and ash layers can hinder reactions, even when ground to less than 10 μm. Under pulsed detonation conditions, anthracite’s rapid heat release promotes detonation wave propagation and stability, achieving the highest efficiency, while peat struggles to sustain combustion and detonation wave propagation due to high ash and low heating value.

## Discussion and prospects

Coal, as a traditional fossil fuel, holds an indispensable position in the energy structure. However, inefficient extraction and high pollution have long been significant challenges. In 2017, Xie et al.^37^ first proposed the “innovative concept of deep in-situ fluidized coal mining,” which integrates multiple technical modules such as excavation, support, coal preparation, energy conversion, and underground energy storage into a Mining Tunnel Boring Machine (M-TBM). The break-through core component of the coal device is the coal powder deflagration unit, designed to convert fossil energy in-situ into electricity or other easily transportable and storable forms of energy through the deflagration of high-purity coal powder obtained from crushing and washing. This innovative approach has paved the way for more efficient and environmentally friendly coal utilization methods.

**Fig. 12:**
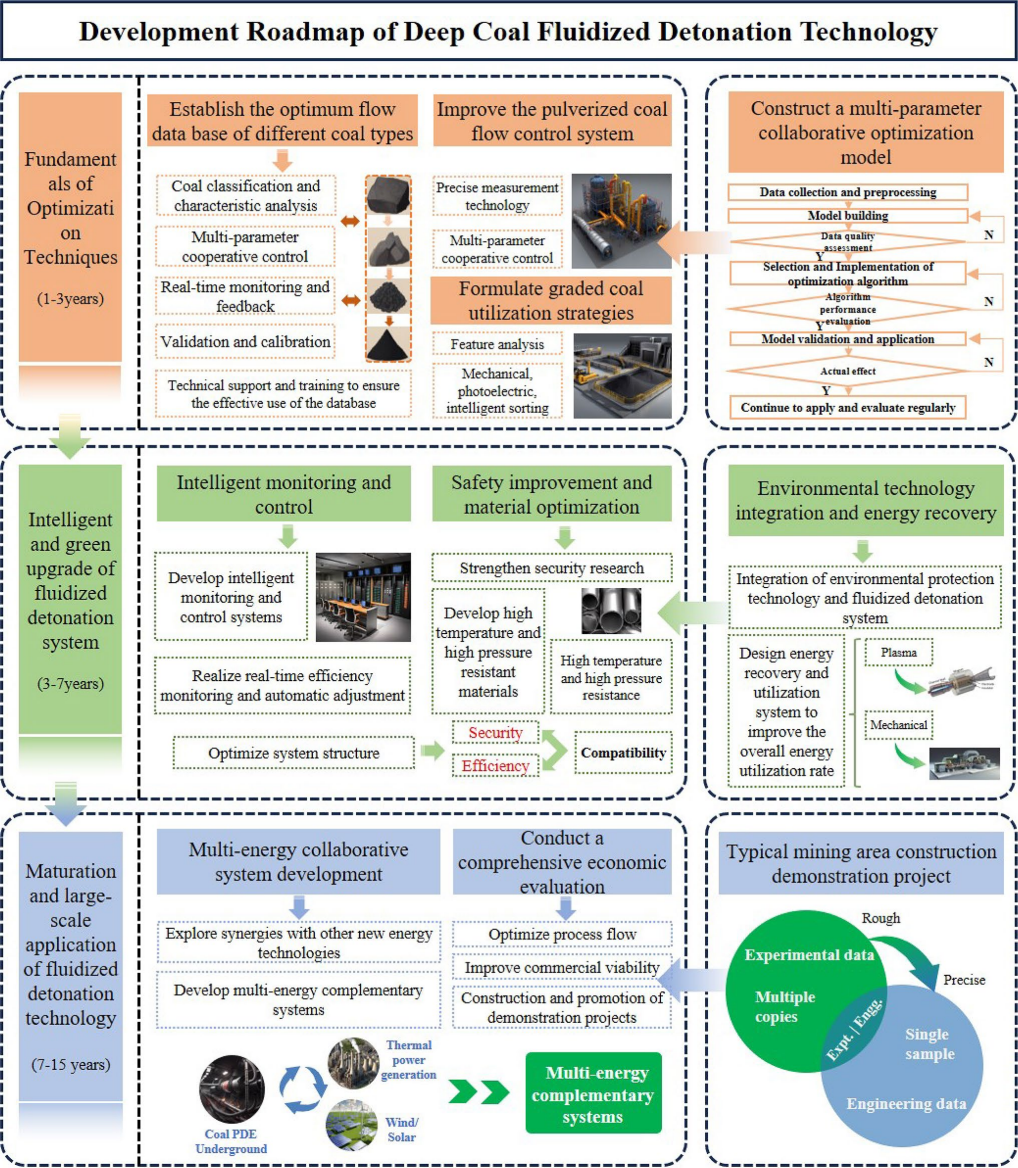
Concept diagram of detonation mechanism system of underground intelligent coal mining.

The introduction of coal powder detonation combustion technology has opened a new technological avenue for efficient coal energy utilization. Compared to deflagration, coal powder detonation technology offers greater adaptability to various coal types and higher combustion efficiency. Despite its immense potential, coal powder detonation combustion technology still faces numerous challenges in practical applications. This study aims to explore the application of coal powder detonation technology in deep coal mining, with a particular focus on the impact of different coal types and coal powder flow rates on detonation efficiency. Based on these research findings, we propose a roadmap for the development of deep coal fluidized detonation technology (As shown in Fig. 12). This roadmap includes short-term, medium-term, and long-term objectives, encompassing various stages from technology optimization to large-scale application.

In the near term (1–3 years), focus should be on optimizing the technological foundation. The primary task is to refine the coal powder flow control system and establish an optimal flow database for different coal types. Simultaneously, develop a strategy for graded coal utilization, prioritizing high-efficiency coal varieties. In-depth research on the impact of parameters like temperature and pressure on efficiency is necessary to construct a multi-parameter synergistic optimization model. In the medium term (3–7 years), efforts should be directed towards system intelligence and environmental protection. Develop intelligent monitoring and control systems to achieve real-time efficiency monitoring and automatic adjustment. Concurrently, research the integration of environmental protection technologies with fluidized detonation systems to ensure clean and efficient mining. We will also initiate the design of energy recovery and utilization systems to improve overall energy efficiency. Safety research will be intensified, developing high-temperature and high-pressure resistant materials and optimizing system structures. In the long term (7–15 years), the goal is to achieve comprehensive technological maturity and large-scale application. Explore synergies with other new energy technologies and develop complementary multi-energy systems. Conduct comprehensive economic assessments, optimize process flows, and enhance commercial viability. Ultimately, construct demonstration projects in typical mining areas to accumulate experience for large-scale promotion.

Through this roadmap, we aim to progressively advance the development of deep coal fluidized detonation technology, ultimately achieving efficient, clean, and safe coal energy exploitation and utilization. This technology not only offers a solution to current challenges in coal mining but also plays a crucial role in the transition towards a more sustainable energy future. As we continue to refine and implement this technology, it has the potential to significantly reshape the landscape of coal utilization, contributing to both energy security and environmental protection.

## Conclusions

This study provides important theoretical and experimental support for the development of pulverized coal detonation technology in deep coal mining. The key findings and conclusions are as follows:

- Detonation pressure (*P*_max_) exhibits a linear increase with coal powder mass flow rate across all tested coal types, with anthracite demonstrating the highest Pmax (1.52 MPa at 120 g/s). A linear relationship between Pmax and maximum temperature (*T*_max_) was also observed for all coal types, indicating consistent thermodynamic behavior during the detonation process.
- The detonation combustion efficiency of pulverized coal (${\eta }_{DCE}$) increases significantly with the rise in coal flow rate. Taking peat as an example, when the coal flow rate increases from 30 g/s to 120 g/s, ${\eta }_{DCE}$ improves from 52.3% to 83.7%. This indicates that increasing the coal flow rate contributes to enhancing combustion efficiency and optimizing the utilization of coal energy.
- The combustion efficiency of different coal types varies. Experimental results indicate that anthracite has the highest combustion efficiency, while peat has the lowest. This is related to the industrial analysis parameters of the coal types, such as ash content, volatile matter, and fixed carbon content. For instance, the lower heating value of anthracite is 31.87 MJ/kg, with an ash content of only 6.79%, whereas the lower heating value of peat is 16.62 MJ/kg, with an ash content as high as 36.01%.
- Based on the research findings, a development roadmap for deep coal fluidized detonation technology has been proposed. This roadmap includes short-term, mid-term, and long-term objectives, aiming to optimize technical parameters, enhance system intelligence, and achieve large-scale application of the technology, providing a new approach for efficient and clean coal utilization.

Future research should focus on further optimizing the coal powder flow control system, developing intelligent monitoring and control systems, and integrating environmental protection technologies. The successful implementation of this technology could significantly reshape the landscape of coal utilization, contributing to both energy security and environmental protection.

## Data Availability

The datasets used and/or analysed during the current study are available from the corresponding author on reasonable request.

## List of symbols

${\eta }_{DEC}$: Detonation combustion efficiency
${C}_{ga}$: Specific heat at constant pressure for gaseous fuel
${C}_{c}$: Specific heat at constant pressure for coal
${C}_{pg}$: Specific heat at constant pressure for gases generated
${S}_{mass}$: Mass source term
${S}_{mom}$: Momentum source term
${S}_{energy}$: Volumetric heat source term
${S}_{species, m}$: Source term produced
${\Delta LHV}_{coal}$: Lower heating value of coal before and after detonation
*A*: Pre-exponential factor
$$R$$: Gas constant
${T}_{i}$: Temperature value at position i
${P}_{i}$: Pressure value at position i
$${K}_{s}$$: P–V characteristic coefficient
$$E$$: Total energy
*E*_*a*_: Activation energy of the reaction
R: Universal gas constant
$$U$$: Velocity vector
$${Y}_{m}$$: Mass fraction
$\Delta t$: Time between adjacent measuring points and detonation point
$$\nabla$$: Hamiltonian operator
*k*: Reaction rate constant
${m}_{a}$: Flow rate of mass
*b*: Temperature exponent
${s}_{m}$: Diffusion flux of component m
$$\mu$$: Dynamic viscosity
$$I$$: Identity tensor
$h$: Total enthalpy of the reactants
$m$: Total number of components
${\dot{\omega }}_{T}$: Rate of chemical reaction
${\dot{\omega }}_{m}$: Rate of production or consumption of component m in reactions
$\tau$: Viscous stress tensor
${v}_{p}$: Detonation wave velocity
${l}_{i+1}$: Distance between adjacent measuring points

## References

[1] Longwell, J. P., Rubin, E. S. & Wilson, J. Coal: Energy for the future. *Prog. Energy Combust. Sci.* **21**(4), 269–360. 10.5860/choice.33-3936 (1995).

[2] Wang, T., Wu, F., Dickinson, D. & Zhao, W. Energy price bubbles and extreme price movements: Evidence from China’s coal market. *Energy Econ.* **129**, 107253. 10.1016/j.eneco.2023.107253 (2024).

[3] Usman, A., Ullah, S., Ozturk, I., Sohail, S. & Sohail, M. T. Does environmental policy stringency reduce trade in energy resources? Insights from coal, petroleum, and gas. *Resour. Policy* **89**, 104679. 10.1016/j.resourpol.2024.104679 (2024).

[4] Wu, H. et al. Complementing carbon tax with renewable energy investment to decarbonize the energy system in China. *Renew. Sustain. Energy Rev.* **189**, 113997. 10.1016/j.rser.2023.113997 (2024).

[5] Li, J. et al. Multifactor configurations of coal power technology in China substantially differ in life-cycle environmental impacts. *Sci. Total Environ.* **907**, 168132. 10.1016/j.scitotenv.2023.168132 (2024). 37890626 10.1016/j.scitotenv.2023.168132

[6] Khatami, R. & Levendis, Y. A. An overview of coal rank influence on ignition and combustion phenomena at the particle level. *Combust. Flame* **164**, 22–34. 10.1016/j.combustflame.2015.10.031 (2016).

[7] Zhang, Z., Zhao, Z. & Zhang, L. Recent progress in the gasification reaction behavior of coal char under unconventional combustion modes. *Appl. Therm. Eng.* **220**, 119742. 10.1016/j.applthermaleng.2022.119742 (2023).

[8] Zhao, S. et al. A review on mercury in coal combustion process: Content and occurrence forms in coal, transformation, sampling methods, emission and control technologies. *Prog. Energy Combust. Sci.* **73**, 26–64. 10.1016/j.pecs.2019.02.001 (2019).

[9] Du, X., Jin, X., Zucker, N., Kennedy, R. & Urpelainen, J. Transboundary air pollution from coal-fired power generation. *J. Environ. Manag.* **270**, 110862. 10.1016/j.jenvman.2020.110862 (2020). 10.1016/j.jenvman.2020.110862 32721309

[10] Putnam, A. A., Belles, F. E. & Kentfield, J. A. C. Pulse combustion. *Prog. Energy Combust. Sci.* **12**(1), 43–79. 10.1121/1.429081 (1986).

[11] Lu, F. K. & Braun, E. M. Rotating detonation wave propulsion: Experimental challenges, modeling, and engine concepts. *J. Propul. Power* **30**(5), 1125–1142. 10.2514/1.B34802 (2014).

[12] Djordjevic, N., Hanraths, N., Gray, J., Berndt, P. & Moeck, J. Numerical study on the reduction of NOx emissions from pulse detonation combustion. *J. Eng. Gas Turbines Power* **140**(4), 041504. 10.1115/1.4038041 (2018).

[13] Meng, X., De Jong, W. & Kudra, T. A state-of-the-art review of pulse combustion: Principles, modeling, applications and R&D issues. *Renew. Sustain. Energy Rev.* **55**, 73–114. 10.1016/j.rser.2015.10.110 (2016).

[14] Blunck, D. L., Apte, S., & Niemeyer, K. Pulse detonation engine for advanced oxy-combustion of coal-based fuel for direct power extraction applications. *Corvallis, OR, US: Oregon State University*. 10.2172/1766796 (2021).

[15] Vasilev, A. A. Characteristics of combustion and detonation of methane-coal mixture. *Combust. Explos. Shock Waves* **49**, 424–434. 10.1134/s0010508213040059 (2013).

[16] Pinaev, A. V. & Pinaev, P. A. Detonation waves in methane/hydrogen/oxygen/coal suspension systems. *Combust. Explos. Shock Waves* **58**(4), 475–480. 10.1134/S0010508222040104 (2022).

[17] Pinaev, A. V. Combustion and detonation waves in methane mixtures with suspensions of fine coal particles. *J. Phys. Conf. Ser.* **1382**(1), 012096. 10.1088/1742-6596/1382/1/012096 (2019).

[18] Pinaev, A. V. & Pinaev, P. A. Combustion and detonation waves in gas mixtures of CH4/Air, CH4/O2, and O2/Coal dust. *Combust. Explos. Shock Waves* **56**(6), 70–681. 10.1134/s0010508220060064 (2020).

[19] Ni, X. et al. Effects of different physical properties of anthracite powder fuel on detonation characteristics of a rotating detonation engine. *Phys. Fluids* **35**(5). 10.1063/5.0149813 (2023).

[20] Debnath, P., & Pandey, K. M. Computational analysis of shrouded ejector effect on starting vortex and combustion efficiency in pulse detonation combustor with different fuels. *Combust. Sci. Technol.*, 1–23. 10.1080/00102202.2023.2280585 (2023).

[21] Debnath, P. & Pandey, K. M. Numerical analysis on detonation wave and combustion efficiency of pulse detonation combustor with U-Shape combustor. *J. Therm. Sci. Eng. Appl.* **15**(10), 101006. 10.1115/1.4062702 (2023).

[22] Debnath, P., & Pandey, K. M. Exergetic and thermal performance analysis of liquid and gaseous fuel–air mixture in PDC using computational fluid dynamics. *Arab. J. Sci. Eng.*, 1–16. 10.1007/s13369-024-09319-5 (2024).

[23] Salvadori, M., Dunn, I. B., Sosa, J., Menon, S., & Ahmed, K. A. Numerical investigation of shock-induced combustion of coal-H2-air mixtures in a unwrapped non-premixed detonation channel. In: *AIAA Scitech 2020 Forum. American Institute Aeronauticsan Astronautics, n. d.*10.2514/6.2020-2159 (2020).

[24] Zhu, W., Wang, Y., & Wang, J. Flow field of a rotating detonation engine fueled by carbon. *Phys. Fluids* **34**(7). 10.1063/5.0099787 (2022).

[25] Zhu, W. & Wang, Y. Effect of hydrogen flow rate and particle diameter on coal-hydrogen-air rotating detonation engines. *Int. J. Hydrogen Energy* **47**(2), 1328–1342. 10.1016/j.ijhydene.2021.10.088 (2022).

[26] Zhu, W., Wang, Y. & Wang, J. Effect of the airmass flow rate on coal-air two-phase rotating detonation waves. *J. Coal Sci.* **47**(7), 3715–3728. 10.1063/5.0099787(inChinese) (2022).

[27] Dunn, I. B., Malik, V., Flores, W., Morales, A. & Ahmed, K. A. Experimental and theoretical analysis of carbon driven detonation waves in a heterogeneously premixed rotating detonation engine. *Fuel* **302**, 121128. 10.1016/j.fuel.2021.121128 (2021).

[28] Zhang, B. et al. The propagation characteristics of particle-laden two-phase detonation waves in pyrolysis mixtures of C (s)/H2/CO/CH4/O2/N2. *Aerosp. Sci. Technol.* **130**, 107912. 10.1016/j.ast.2022.107912 (2022).

[29] Shi, J., Zhang, P., Xu, Y., Ren, W. & Zhang, H. Effects of dilute coal char particle suspensions on propagating methane detonation wave. *Combust. Flame* **249**, 112618. 10.1016/j.combustflame.2023.112618 (2023).

[30] Bykovskii, F. A. E., Vedernikov, E. F. & Zholobov, Y. A. Detonation combustion of lignite with titanium dioxide and water additives in air. *Combust. Explos. Shock Waves* **53**, 453–460. 10.1134/s0010508217040098 (2017).

[31] Bykovskii, F. A. E., Zhdan, S. A., Vedernikov, E. F. & Zholobov, Y. A. Detonation burning of anthracite and lignite particles in a flow-type radial combustor. *Combust. Explos. Shock Waves* **52**, 703–712. 10.1134/s0010508216060101 (2016).

[32] Bykovskii, F. A. E., Zhdan, S. A., Vedernikov, E. F. & Zholobov, Y. A. Detonation of a coal-air mixture with addition of hydrogen in plane-radial vortex chambers. *Combust. Explos. Shock Waves* **47**, 473–482. 10.1134/s0010508211040113 (2011).

[33] Bykovskii, F. A. E., Zhdan, S. A., Vedernikov, E. F. & Zholobov, Y. A. Detonation combustion of coal. *Combust. Explos. Shock Waves* **48**, 203–208. 10.1134/s0010508212020098 (2012).

[34] Bykovskiĭ, F. A., Zhdan, S. A., Vedernikov, E. F. & Zholobov, Y. A. Continuous and pulsed detonation of a coal-air mixture. *Dokl. Phys.* **55**(3), 142–144. 10.1134/S1028335810030092 (2010).

[35] Xu, G. et al. Characterization of wave modes in a kerosene-fueled rotating detonation combustor with varied injection area ratios. *Appl. Therm. Eng.* **212**, 118607. 10.1016/j.applthermaleng.2022.118607 (2022).

[36] Roy, G. D., Frolov, S. M., Borisov, A. A. & Netzer, D. W. Pulse detonation propulsion: Challenges, current status, and future perspective. *Prog. Energy Combust. Sci.* **30**(6), 545–672. 10.1016/j.pecs.2004.05.001 (2004).

[37] Xie, H., Ju, Y., Gao, F., Gao, M. & Zhang, R. Groundbreaking theoretical and technical conceptualization of fluidized mining of deep underground solid mineral resources. *Tunn. Undergr. Space Technol.* **67**, 68–70. 10.1016/j.tust.2017.04.021 (2017).

[38] Guo, J., Ge, S., Guo, Y., Liang, J. & Yang, R. Study on detonation characteristics of pulverized coal and evolution law of detonation residue. *Sci. Rep.* **14**(1), 11691. 10.1038/s41598-024-62489-y (2024). 38778094 10.1038/s41598-024-62489-y PMC11111701

[39] Guo, J., Ge, S., Guo, Y., Liang, J. & Yang, R. Experimental study and application prospects of coal dust/methane/oxygen pulse detonation performance: Coal dust equivalence ratio and types. *ACS Omega* **9**(33), 35287–35300. 10.1021/acsomega.3c10511 (2024). 39184517 10.1021/acsomega.3c10511 PMC11339806

